# A Genomewide Screen for Suppressors of *Alu*-Mediated Rearrangements Reveals a Role for PIF1

**DOI:** 10.1371/journal.pone.0030748

**Published:** 2012-02-09

**Authors:** Karen M. Chisholm, Sarah D. Aubert, Krister P. Freese, Virginia A. Zakian, Mary-Claire King, Piri L. Welcsh

**Affiliations:** 1 Department of Genome Sciences, University of Washington, Seattle, Washington, United States of America; 2 Department of Medicine, Division of Medical Genetics, University of Washington, Seattle, Washington, United States of America; 3 Department of Molecular Biology, Princeton University, Princeton, New Jersey, United States of America; Kyushu Institute of Technology, Japan

## Abstract

*Alu*-mediated rearrangement of tumor suppressor genes occurs frequently during carcinogenesis. In breast cancer, this mechanism contributes to loss of the wild-type *BRCA1* allele in inherited disease and to loss of heterozygosity in sporadic cancer. To identify genes required for suppression of *Alu*-mediated recombination we performed a genomewide screen of a collection of 4672 yeast gene deletion mutants using a direct repeat recombination assay. The primary screen and subsequent analysis identified 12 candidate genes including *TSA*, *ELG1*, and *RRM3*, which are known to play a significant role in maintaining genomic stability. Genetic analysis of the corresponding human homologs was performed in sporadic breast tumors and in inherited *BRCA1*-associated carcinomas. Sequencing of these genes in high risk breast cancer families revealed a potential role for the helicase PIF1 in cancer predisposition. PIF1 variant L319P was identified in three breast cancer families; importantly, this variant, which is predicted to be functionally damaging, was not identified in a large series of controls nor has it been reported in either dbSNP or the 1000 Genomes Project. In *Schizosaccharomyces pombe*, *Pfh1* is required to maintain both mitochondrial and nuclear genomic integrity. Functional studies in yeast of human PIF1 L319P revealed that this variant cannot complement the essential functions of *Pfh1* in either the nucleus or mitochondria. Our results provide a global view of nonessential genes involved in suppressing *Alu*-mediated recombination and implicate variation in *PIF1* in breast cancer predisposition.

## Introduction

*Alu* elements account for more than 10% of the human genome [1] and consequently provide abundant opportunities for unequal homologous recombination both intrachromosomally, resulting in deletion or duplication of exons in a gene, and interchromosomally, causing more complex chromosomal abnormalities. Thus, it is not surprising that unequal homologous recombination between *Alu* repeats contributes to a significant proportion of human genetic disease [2].

Germline mutations in *BRCA1* predispose to breast and ovarian cancer. The 84-kb *BRCA1* locus is densely packed with repetitive elements including 138 individual *Alu* repeats that comprise 41.5% of the total sequence [3]. While the majority of known *BRCA1* mutations are small nucleotide sequence alterations (Breast Cancer Information Core database, http://research.nhgri.nih.gov/bic), mutations involving *Alu* sequences are common. To date, at least 81 large genomic rearrangements in *BRCA1* have been identified in high-risk breast cancer families, the majority of which are deletions ranging in size from a few hundred base pairs, to tens of kilobases. Of these, 59 are due to *Alu*-mediated unequal homologous recombination [4]. Of the remaining characterized deletions, 16 are the result of nonhomologous recombination events, eight of which involve one *Alu* repeat at either the 5′ or 3′ breakpoint, and five are the result of recombination between *BRCA1* and the human *BRCA1* pseudogene. Importantly, large genomic rearrangements account for up to 12% of all novel *BRCA1* mutations identified in high-risk breast cancer families [5].

LOH at 17q has been detected in about 30%–60% of sporadic breast tumors and, in many instances, includes the *BRCA1* locus [6]–[9]. Loss of heterozygosity at the *BRCA1* locus has been reported in 20%–70% of sporadic breast and ovarian cases [10]–[14] and in breast cancers has been correlated with larger tumor size, higher grade, and negative hormone receptor negative status [15]. Thus, it is possible that *Alu*-mediated recombination in may be responsible for a significant proportion of germline deletions and/or rearrangements in *BRCA1* as well as contribute to allelic loss of *BRCA1* in sporadic disease. However, the genes responsible for suppressing *Alu*-mediated genomic instability remain unknown.

Previously, our lab exploited a functional assay in yeast to search for mutations caused by deficiencies of the yeast homologs of human mismatch repair genes [16]. These studies demonstrated that yeast is a model organism for examining mutation rates in known human tumor suppressor genes. We modified this approach to investigate the mechanisms underlying *Alu*-mediated unequal homologous recombination. Here we report the identification of yeast genetic backgrounds permissive for high frequency *Alu*-mediated rearrangement at the *BRCA1* locus. Because many genes responsible for maintaining genomic stability are highly conserved evolutionarily across species including yeast and mammals [17], [18], we characterized variation in the human homologs of the yeast genes to determine whether mutation contributes to either inherited and/or sporadic breast tumorigenesis. Our global analysis of non-essential genes involved in suppressing *Alu*-mediated recombination has identified human genes not previously known to be involved in maintaining genomic stability. We present both genetic and functional data which suggests that PIF1 may function as a tumor suppressor.

## Materials and Methods

### Plasmid construction

**Figure 1:**
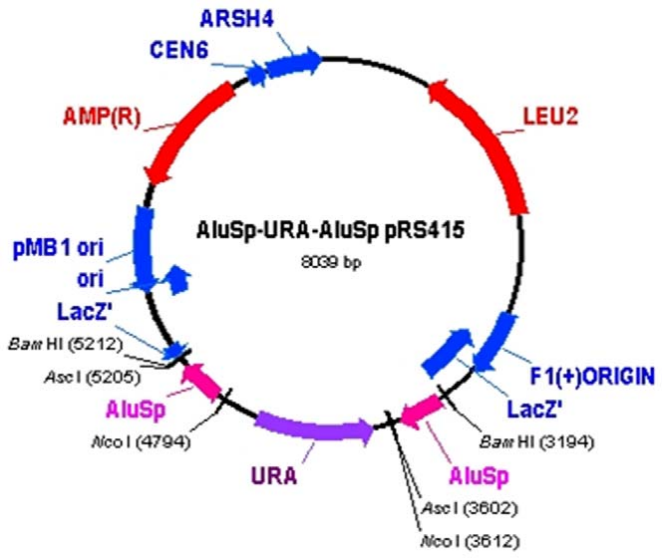
*Alu*-URA-*Alu* pRS415 (pAUA). A construct containing a *URA3* gene flanked by identical *AluSp* repetitive elements was inserted into the low-copy-number yeast plasmid pRS415.

Primer sequences used for cloning purposes are listed in Table S1. The *BRCA1* intron 16 *AluSp* element (Genbank L78833; 58500–58798), was amplified from genomic DNA and cloned into plasmid pCR®2.1-TOPO® using the TOPO TA Cloning® protocol (Invitrogen). Primer design incorporated restriction enzyme target sequences for BamHI, AscI and NcoI to facilitate cloning into pRS415, a centromeric vector with a *LEU2* marker. Following site-directed mutagenesis (GeneEditor™ in vitro Site-Directed Mutagenesis System; Promega) of an internal Nco1 site in the *URA3* gene from plasmid pRS416, the gene was amplified with primers which incorporated flanking NcoI restriction enzymes sites. Construction of *Alu*-URA-*Alu* pRS415 proceeded as follows: the BamHI-flanked *Alu* element was ligated into pRS415, followed by the AscI-flanked *Alu* element, and lastly, the *URA3* gene was cloned between the *AluSp* elements using the engineered NcoI restriction sites. *Escherichia coli* TOP10 One Shot® competent cells were used for initial bacterial transformations while SURE® (Stop Unwanted Rearrangement Events) competent cells were used for transformation and propagation of the final construct, pAlu-URA-Alu (pAUA), Figure 1, which was sequenced to confirm presence and orientation of inserts.

### Functional screen for suppressors of *Alu*-mediated recombination

The collection of haploid yeast deletion strains screened in this study has been previously described [19]. All strains were propagated at 30°C. Deletion strains were transferred using a multipronged replica-plating device to 96-well plates containing 100 microliters of standard YPAD media supplemented with 10 mg/ml G418 and grown overnight. 30 microliters of each culture was transferred to a fresh plate containing 80 microliters of YPAD. After a five hour incubation period, yeast were transformed with 200 ng of pAUA plasmid DNA using the lithium acetate/PEG method [20]–[22]. Following transformation, each well was individually plated onto selective medium (SC –Leu/−Ura) and incubated at 30°C. After three days of growth, individual colonies from each transformation were picked with a sterile toothpick and diluted in 100 microliters dH_2_O. A total of 8 microliters of dilution culture was streaked onto SC –Leu/+5FOA plates. Deletion of the *URA3* gene in pAUA is permissive for growth on 5FOA. Following a 3-day incubation, 5-FOA^R^ was scored as follows: 0 colonies = 0; 1–5 colonies = 1; 6–10 colonies = 2; 11–15 colonies = 3; 16–34 colonies = 4; ≥35 colonies = 5 (Figure S1). This screen was repeated and duplicate scores were added together to generate an overall score for each deletion strain of 0–10. Naïve strains with scores of 7–10 were retransformed and re-screened. Suppressors of *Alu*-mediated recombination identified in the BY4742 background were re-verified by analyzing their counterparts in the BY4741 background. Identifying tag sequences for stains were determined and compared to the tag lists on http://www-sequence.stanford.edu/group/yeast_deletion_project/strain_alpha_mating_type.txt or http://www-sequence.stanford.edu/group/yeast_deletion_project/strain_a_mating_type.txt. We acknowledge that this screen would fail to reveal human genes involved in suppressing *Alu*-mediated rearrangements for which there are no homologs in budding yeast.

### Fluctuation analysis

The *Alu*-mediated recombination rate for individual yeast deletion strains was determined by fluctuation analysis using the method of the median [23] as previously described [24], [25]. In brief, yeast successfully transformed with plasmid pAUA were selected on SC –Ura/−Leu plates. For each fluctuation analysis, ten individual colonies from each strain were each resuspended in water and dilutions were plated on −Leu^/^+5FOA plates (to measure 5-FOA^R^) and −Leu plates (to monitor viable cells). The number of colonies on each plate was counted after three days of growth at 30°C. Mutation rates represent 5-FOA^R^ events/cell division. For each strain, fluctuation analysis was performed independently three to four times with final mutation rates determined by averaging individual results. Student's t-test was used to calculate p-values.

### Human homolog identification

The entire yeast amino acid sequence for each gene was evaluated using BLASTp (http://www.ncbi.nlm.nih.gov/BLAST/). To be considered the human homolog of the respective yeast gene, human genes had to have an e value greater than e-28. Genomic sequences of human homologs were obtained using the University of Santa Cruz Genome Database (http://www.genome.ucsc.edu).

### Patients and control samples

Human DNA samples evaluated in this study were from (a) a cohort of 44 Ashkenazi Jewish probands from families with at least four cases of breast cancer and no known mutations in the high risk breast cancer genes *BRCA1*, *BRCA2*, and *CHEK2* [5], (b) a cohort of 94 women with known *BRCA1* mutations who developed breast cancer at or before the age of 39 and/or ovarian cancer at or before the age of 64 [5], (c) a series of 196 Ashkenazi Jewish controls, (d) a series of 200 Caucasian controls, (e) a series of an additional 900 Ashkenazi Jewish breast cancer cases [5], and (f) a series of 400 additional high risk breast cancer probands [5]. All cancer diagnoses were verified by pathology reports and/or hospital records. The study was approved by the University of Washington Human Subjects Division (IRB protocol 34173). All participants provided informed consent.

### Loss of heterozygosity of candidate genes in sporadic breast cancers

Tumor specimens were obtained from the Cooperative Human Tissue Network. Microsatellite markers flanking each human homolog were chosen using the University of Santa Cruz Genome Database (http://www.genome.ucsc.edu). Table S2 lists the microsatellite markers used to evaluate loss of heterozygosity in a series of 25 sporadic grade III breast tumors. Haematoxylin and eosin stained sections from formalin-fixed paraffin-embedded tissues were reviewed prior to DNA extractions. Tumor DNA from regions displaying greater than 70% neoplasticity and normal DNA was extracted using the PicoPure™ DNA Extraction Kit (Arcturus Bioscience). PCR reactions included 500 microCi [alpha-^32^P] dCTP. PCR products were separated by capillary electrophoresis using 6% polyacrylamide gels. The gels were dried under vacuum and exposed to x-ray film. For all heterozygous alleles, LOH was defined as loss of ≥50% radioactive intensity in tumor DNA compared to normal.

### Genomic sequencing

Sequencing primers were designed using the MacVector software (version 7.2) so that all exonic and at least 20 base pairs of flanking intronic sequence were evaluated. Primer sequences are available upon request. Total genomic DNA was extracted from Epstein-Barr virus-immortalized lymphoblast cell lines using the Puregene DNA extraction system (Gentra). PCR reactions consisted of 100 ng of genomic DNA, 10 pmole of each primer, 200 microM of dATP, dTTP, and dGTP, dCTP, 1X PCR Buffer (Invitrogen), 1.5 mM MgCl_2_, and 1 unit Taq polymerase (Invitrogen). PCR products were purified and then bidirectionally sequenced on either an ABI 3100 or ABI 3730 DNA Analyzer. Resulting DNA sequence was analyzed using either Sequencing Analysis (version 3.3) or Sequencher™ (version 4.2).

### Substitution tolerance and protein structure prediction

Amino acid substitutions were characterized with two computer resources to predict if they were deleterious: SIFT (Sorting Intolerant from Tolerant) (http://blocks.fhcrc.org/sift/SIFT.html or http://sift.jcvi.org/sift-bin) and PolyPhen (Polymorphism Phenotyping) (http://genetics.bwh.harvard.edu/pph/). Secondary structure elements were predicted using PSIPRED (http://bioinf.cs.ucl.ac.uk/psipred/) and PredictProtein (http://www.predictprotein.org/) programs.

### Cross complementation assays in *Schizosaccharomyces pombe*

Strain genotypes are listed in Table S3. *Schizosaccharomyces pombe* (*S. pombe*) cells were cultured in supplemented yeast extract (YES, Difco) or supplemented Edinburgh minimal medium [26] at 32°C, unless otherwise noted. Leucine 430 (codon TTA) was mutated to proline (codon CCG) by site-directed mutagenesis (Stratagene) on a vector carrying a *leu^+^* marker. Using a rapid transformation method [27], *pfh1-L430P* was integrated at the *leu1-32* locus of a strain containing *loxP pfh1^+^ kanMX6 loxP* at the endogenous *pfh1* locus. Strains were grown to log phase in supplemented liquid Edinburgh minimal medium. A rapid transformation protocol [27] was used to introduce either pREP82 *cre* (*cre^+^*) or pREP82 *cre (Y324F)* (*cre^−^*) [28]. The Y324F point mutation abolishes catalytic activity of Cre recombinase which promotes recombination at loxP sites. Transformation plates were typically incubated at 32°C for 4 days except plates with pfh1-mt* which were incubated at 18°C for 6 days and 30°C for 3 days. For western blot analysis, *pfh1-L430P* was integrated at the *leu1-32* locus of a strain containing *nmt 81 pfh1^+^ GFP* at the endogenous *pfh1* locus. Pfh1-GFP was depleted by the addition of 30 µM thiamine over 24 hours. Whole cell extracts were prepared by glass bead lysis in HB buffer [26], containing a protease inhibitor cocktail (Roche). Protein concentration was determined by BCA protein assay kit (Pierce) and 200 µg of total protein was loaded onto a 7.8% SDS-PAGE. Blots were probed with rabbit anti-Pfh1 polyclonal serum [29] and HRP-conjugated goat anti-rabbit IgG polyclonal serum (BioRad).

## Results

### A genome-wide screen in *S. cerevisiae* for suppressors of *Alu*-mediated rearrangements

There are 11 subfamilies of *Alu* elements in the *BRCA1* gene that differ in sequence and in length [3]. The pAUA construct was created using a *BRCA1 AluSp* sequence (Genbank L78833 58500–58798) as this subfamily has been shown to be frequently involved in *BRCA1* genomic rearrangements. The *URA3* gene was inserted between these *Alu* elements to allow for selection. The resulting plasmid (pAUA, Figure 1) was transformed into the complete set of tagged deletion strains from the *Saccharomyces cerevisiae* Genome Project. A direct repeat recombination assay marked by loss of *URA3* expression was performed with *URA3* deficient strains identified by their ability to grow on plates containing the drug 5-FOA. Resistance to this drug (5-FOA^R^) was scored as described in Materials and Methods (Figure S1). The total collection contains 4672 deleted strains in the BY4742 background of which 4634 (99.2%) were successfully screened using this approach. Results of this screen are summarized in Table S4. Overall, 548 strains (11.8%) had a score of 8 or greater. Naïve yeast from these 548 deletion strains were retransformed with pAUA and 5-FOA^R^ colonies scored. Mutation rates for the 66 deletion stains with scores of 7 or higher were determined by fluctuation analyses and compared to the parental strain mutation rate. Table 1 lists the 12 yeast deletion strains with *Alu*-mediated rearrangement rates of greater than 1.5 fold over that of wild-type. Mutation rates ranged from 1.5 to 10.6 fold higher than that of the wild-type mutation rate. Deletion cassette tags for these strains were sequenced to confirm strain assignment.

**Table 1:** *Alu*-mediated recombination rates and genetic analyses of candidate suppressors of *Alu* mediated recombination.

*Yeast Gene*	*Yeast ORF*	*Mutation Rate ×10^−6^*	*Fold Induction over WT*	*p-value*	*Human Homolog(s)*	*BRCA1 modifiers* a	*LOH* b	*AJ screen* c	*Additional screening* d
ARG3	YJL088W	45.0	10.61	0.055	OTC	No Mutations	6/20	No Mutations	
ELG1	YOR144C	19.5	4.60	0.041	none				
TSA1	YML028W	14.2	3.34	0.001	PRDX1 PRDX2 PRDX3 PRDX4	No Mutations No Mutations L181H IVS3+3C/T	6/23 2/25 4/24 3/20	No Mutations No Mutations No Mutations No Mutations	
RRM3	YHR031C	12.0	2.82	0.018	PIF1 (C15orf20)	V21L, P109S	2/22	L319P	S223T, D314E, P357L, R592C
orf	YPR170C	11.8	2.79	0.079	none				
OMA1	YKR087C	10.5	2.47	0.190	OMA1	D365N	0/25	No Mutations	
MPH1	YIR002C	7.89	1.86	0.009	FANCM	T176M, C182S, N689S, Q1730P, I1742V, V2014A	3/22	H1703R, I1742V	
HSM3	YBR272C	7.89	1.86	0.010	none				
REC104	YHR157W	7.34	1.73	0.0009	none				
REC8	YPR007C	7.13	1.68	0.218	none				
GOR1	YNL274C	7.04	1.66	0.145	GRHPR				
TEL1	YBL088C	6.44	1.52	0.054	ATM				
**Wild-type**	BY4742	4.24	1.00						

^*a*^Unreported heterozygous mutations within each gene screened for in women with early breast and/or ovarian cancer and with *BRCA1* mutations.

^*b*^Number of sporadic breast tumors with loss of heterozygosity (LOH) of markers within or near each gene.

^*c*^Unreported heterozygous mutations within each gene screened for in Ashkenazi Jewish families with breast cancer but without mutations in the known breast cancer genes.

^*d*^Unreported heterozygous mutations within *PIF1* in a series of 400 additional high-risk breast cancer probands with no mutations in the known breast cancer genes.

To determine whether loss of URA3 expression was the result of *Alu*-mediated recombination between direct repeats as opposed to other mechanisms (e.g. point mutation), pAUA plasmids were rescued from 5-FOA^R^ colonies from strains with mutation rates greater than 1.5 fold that of wild-type. In all 12 strains, sequencing of rescued plasmids revealed constructs that retained only one *Alu* element, confirming that the mechanism responsible for loss of URA3 expression was deletion of genomic sequence via *Alu*-mediated rearrangement.

To validate initial observations, the corresponding gene deletion strains of the twelve potential mutator genes in the BY4741 background were transformed with the reporter plasmid and fluctuation analyses performed. Mutation rates were calculated and compared to wild-type BY4741. Mutation rates for strains YJL088W (*arg3*), YHR031C (*rrm3*), YML028W (*tsa1*), and YKR087C (*oma1*) were greater than 1.5 fold that of wild-type (Table S5).

### Identification of human homologs for yeast genes that suppress *Alu*-mediated unequal homologous recombination

In order to identify potential human homologs of yeast genes, amino acid sequences corresponding to the deleted yeast genes were compared using BLASTp to all *Homo sapiens* proteins. Table 1 lists the potential human homologs, and Table S6 provides their BLASTp e-values, human chromosomal locations, and a brief description of function for each protein. Note that yeast *Tsa1* has four potential human homologous: *PRDX1*, *PRDX2*, *PRDX3*, and *PRDX4*. As expected, some yeast genes did not have an identifiable human homolog. Human homologs corresponding to yeast genes with mutation rates above 1.8 fold in *BY4742* deletion strains were used in subsequent studies. These human genes were: *OTC*, *PRDX1*, *PRDX2*, *PRDX3*, *PRDX4*, *PIF1*, *OMA1*, and *FANCM*.

### Novel variants in suppressors of *Alu*-mediated recombination do not modify *BRCA1*-associated cancer risk

Among carriers of *BRCA1* mutations, there is significant variability in the age of onset of cancer [30]–[32]. Studies of high-risk families quantifying the extent of risk variation have suggested that other genetic factors may modify the risk of breast cancer associated with *BRCA1* mutations [33]–[35]. Importantly, it is likely that initiation of a significant proportion of neoplastic transformation in mutation carriers is the result of *Alu*-mediated aberrant homologous recombination events resulting in somatic loss of the wild-type allele. In order to determine if variation in candidate genes modifies the effect of germline mutation in *BRCA1*, women with inherited mutations in *BRCA1* who developed breast cancer at or before the age of 39 and/or ovarian cancer at or before the age of 64 were identified from a large series of *BRCA1* mutation carriers. Genomic sequence for the eight candidate genes was determined using DNA from the selected 94 *BRCA1* mutation carriers. Polymorphisms listed on the UCSC Genome Browser as known SNPs or reported in the 1000 Genomes Project were not considered further. Eleven novel heterozygous variants in these human homologs were identified (Table 1 and Table S7): one in PRDX3, one in PRDX4, two in PIF1, one in OMA1, and six in FANCM. No novel variants were identified in OTC, PRDX1, or PRDX2. The novel protein variants in PRDX3 and OMA1 were predicted to be benign by Polyphen and SIFT, computational tools used to predict if amino acid substitutions are deleterious. All six novel variants in FANCM were also predicted to be benign by SIFT and PolyPhen; in addition two of these mutations were identified in controls. The V21L mutation in PIF1 was also predicted by PolyPhen and SIFT to be benign. The P109S mutation in PIF1 was predicted to be probably damaging but was identified in control populations. We also determined whether, in a family in which a respective variant was found, whether **all** BRCA1 mutation carriers in a family also carried the respective variant allele. However, no variants segregated with BRCA1 carrier status in a given family. Thus, we conclude that these variants did not modify risk of breast and/or ovarian cancer in BRCA1 mutation carrier.

### Genomic analysis of suppressors of *Alu*-mediated recombination in sporadic breast tumors

Tumor suppressor genes often show evidence of heterozygous gene loss in cancers. For example, loss of heterozygosity (LOH) at *BRCA1* is common in both inherited and sporadic breast cancer. In order to determine if any of the genes identified in this screen are candidate breast cancer tumor suppressor genes, twenty-five sporadic breast tumors were analyzed for LOH at the respective candidate gene loci. Two microsatellite markers for each gene (Table S2) were amplified using tumor and corresponding normal DNA. Results are listed in Table 1 (and shown in Figure S2). Of the loci evaluated for LOH in breast tumors, the *Arg3* homolog, *OTC*, displayed LOH in 30% of breast tumors, while the *Tsa1* homolog, *PRDX1*, was lost in 26% of tumors. For each tumor that displayed LOH, the gene of interest was sequenced in order to identify inactivating mutations on the retained allele. However, no inactivating somatic mutations were identified, suggesting that *PRDX1* and *OTC* do not play a role in sporadic breast tumorigenesis.

### Analysis of suppressors of *Alu*-mediated recombination in probands from high-risk breast cancer families

In order to determine if any of the genes revealed in our screen contributed to increased risk in unexplained high-risk breast cancer families, defined as families with 4 or more cases of breast or ovarian cancer, we chose to study a population where genetic heterogeneity is reduced. Genomic DNA from probands of 44 Ashkenazi Jewish high-risk breast cancer families (wild-type for *BRCA1*, *BRCA2* and *CHEK2*) was sequenced. Polymorphisms listed on the UCSC Genome Browser, dbSNP, or 1000 Genomes were not considered to be new mutations. Three previously unreported substitutions were identified, two in the *FANCM* gene, and one in the *PIF1* gene (Table 1 and Table S8). The mutations in *FANCM* were identified in the control series and thus were not considered to contribute to elevated breast cancer risk. However, the heterozygous PIF1 variant L319P was not found in a series of 184 Ashkenazi Jewish controls or in 184 Caucasian controls. Both SIFT and PolyPhen indicated that this substitution was likely to be deleterious. This variant was identified in a family in which the proband had breast cancer at the age of 37. The substitution was inherited from the proband's father who was diagnosed with prostate cancer at 60. The proband's paternal grandmother also carried the variant and was diagnosed with breast cancer at 63. Interestingly, her paternal aunt who also carried the variant was diagnosed with extramammary Paget's disease in the genital area. This condition is an exceedingly rare intraepithelial adenocarcinoma and is often associated with neoplasms arising in the bladder, urethra, or prostate (reviewed in [36]).

Because this family is of Ashkenazi Jewish ancestry, we screened normal DNA from an additional 844 Ashkenazi Jewish breast cancer cases for PIF1 L319P and identified two probands who carried this allele. Of note, PIF1 L319P was not detected in almost 10,000 chromosomes evaluated in the Exome Variant series (http://evs.gs.washington.edu/EVS). We also determined the sequence of the entire *PIF1* coding sequence and 20 bp of flanking intronic sequence in normal DNA from 400 additional high-risk breast cancer probands from of European ancestry. For all probands, *BRCA1* and *BRCA2* had been determined to be wildtype on the basis of commercial sequencing and BART analysis by Myriad Genetics [37]. Although PIF1 variant L319P was not observed in this series, four novel heterozygous variants were identified (Table 2). All PIF1 variants identified to date are listed in Table 2. Of the additional variants, only the S223T and R592C substitutions were predicted to be intolerable by both SIFT and PolyPhen.

**Table 2:** PIF1 variants.

*Heterozygous Mutation*	*# of families*	*Frequency in controls*	*SIFT* a *del?* b *align.* c		*PolyPhen score Δ* d predictione **		*1000 Genomes*	*dbSNP*	*Exome Variant*	*Secondary Structure* g *prediction confidence*	
V21L	1	0/200^h^	0.16	0.41	1.465	Benign	Not present	Not present	Not present	Strand	9
P109S	1	1/198^h^	0.05	0.49	2.506	Probably damaging	Not present	Not present	0.005 EA 0.001 AA	Coil	9
S223T	1		0.01	0.96	1.544	Possibly damaging	Not present	Not present	0.001 EA 0.0003 AA	Coil	1
D314E	1		0.42	0.98	0.366	Benign	Not present	Not present	Not present	Helix	8
L319P	3	0/184^h^ 0/184^i^	0.00	0.99	2.139	Probably damaging	Not present	Not present	Not present	Helix	9
P357L	1		0.18	0.98	1.716	Possibly damaging	Not present	Not present	0.0000 EA 0.002 AA	Coil	9
R592C	1		0.01	0.99	2.913	Probably damaging	R592H	R592H; rs114090726	0.0004 EA 0.0000 AA	Strand	9

a PIF1: gi for SIFT prediction is 82546872; protein identifier for PolyPhen prediction is Q330H5.

b SIFT prediction probability of deleterious allele (<0.05 is deleterious).

c SIFT alignment score (1.00 is highest).

d PolyPhen Position-Specific Independent Counts (PSIC) profile score difference (large values may indicate that the studied substitution is rarely or never observed in the protein family).

e PolyPhen prediction.

f Exome variant database: EA = European Americans; AA = African Americans.

g Predicted secondary structure using PSIPRED (confidence level 0 = low; 9 = high).

h Caucasian control group.

i Ashkenazi Jewish control group.

### Bioinformatic analysis of human PIF1 variant L319P

Protein alignment with PIF1 homologs places PIF1 L319 between conserved helicase motifs II and III [38]. Amino acid L319 is 93% conserved with only one noted conservative substitution in *Chlamydia muridarum*. To determine if the PIF1 L319P variant impacts protein structure, bioinformatic resources were used to evaluate conservation of the amino acid in which the substitution occurred. The PSIPRED protein secondary structure prediction program predicted with a high confidence level that amino acid L319 is in the middle of a helix.

### Functional analysis of human PIF1 variant L319P in yeast

*S. pombe Pfh1* is required to maintain both mitochondrial and nuclear genome integrity [39]. The *S. pombe Pfh1* allele L430P corresponds to the *H. sapiens* L319P allele. To determine if a *pfh1*-L430P allele could provide the essential function of *pfh1^+^* in *S. pombe*, three different strains were constructed (Table S3). All three strains had a wild-type (WT) *pfh1^+^* flanked by loxP sites at its endogenous locus. When WT Cre recombinase was introduced into these strains, it stimulated recombination between the two loxP sites flanking *pfh1+*, resulting in a *pfh1* cell. The strains also carried either *pfh1*-L430P, wild-type *pfh1^+^*, or an empty vector integrated at the *leu1-32* locus. The ectopic copies of mutant or WT *pfh1* were expressed from the *pfh1^+^* promoter. Cre recombinase was introduced into the three strains by introducing a plasmid containing cre^+^ and *ura4* ^+^. In addition, the three strains were independently transformed with a plasmid containing *ura4* ^+^ and a catalytically inactive Cre recombinase (cre^−^), which served as a control for transformation efficiency [28].

**Figure 2:**
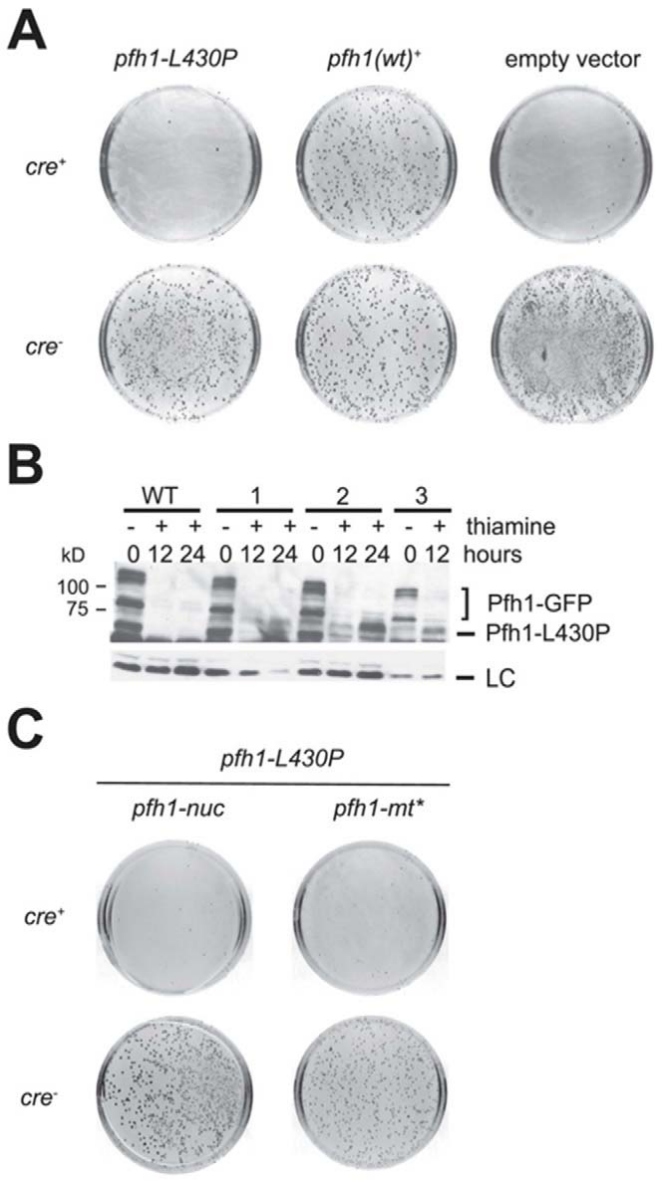
*pfh1-L430P* does not complement wild-type *pfh1^+^* function. (**A**) Complementation assay in *pfh1::loxP pfh1^+^ kanMX6 loxP* strain with either the L430P mutation (*leu::pfh1-L430P*), Pfh1 (*leu::pfh1^+^*), or an empty vector (*leu::leu^+^*) strain after transformation with a plasmid expressing Cre (*cre^+^*) or Cre-Y324F (*cre* ^−^). The transformation plates are contrast-inverted pictures. (**B**) Anti-Pfh1 western blot of wild-type Pfh1GFP (WT) and three independent clones (1–3) expressing Pfh1-L430P. *S. pombe* cells were grown in the absence (−) or presence (+) of thiamine. 0, 12, and 24 hours indicates the amount of time cells were grown in the presence of thiamine. The loading control (LC) is a non-specific background band. (**C**) Heterologous expression constructs of *pfh1-nuc* or pfh1-mt* are indicated in the presence of *pfh1::loxP pfh1^+^ kanMX6 loxP* and *leu::pfh1-L430P*. Contrast-inverted pictures of transformation plates with *cre^+^* on the top and *cre^−^* on the bottom.

Strains expressing *pfh1-L430P* produced very few Ura^+^ colonies when transformed with *cre^+^* (Figure 2A, top row left). However, a large number of Ura^+^ transformants were observed with the catalytically inactive *cre^−^* control (Figure 2A, second row left). Similar results were seen with the negative control strain in which very few Ura^+^ transformants were seen with an empty vector at the *leu1-32* locus, and very few Ura^+^ colonies were obtained with the *cre^+^* plasmid and many Ura^+^ cells with the *cre^−^* control (Figure 2A first and second rows right). In contrast, in the positive control strain having *pfh1^+^* at *leu1-32*, large numbers of Ura^+^ transformants were observed after transformation with either *cre^+^* or *cre^−^* recombinase plasmids (Figure 2A first and second rows middle). Further analysis of the resulting colonies from the *cre^+^* transformation of the *pfh1-L430P* and empty vector revealed that they were kanamycin resistant, indicating that wild-type Pfh1 was expressed in these transformants due to lack of recombination at the loxP sites.

The *pfh1-L430P* allele could fail to support viability either because the mutant protein is non-functional or because it is not stably maintained. To distinguish between these possibilities, strains expressing GFP-tagged *Pfh1* at its endogenous locus and *Pfh1*-L430P at the *leu1-32* locus were employed (Table S3). Since degradation products from Pfh1-GFP overlapped with the Pfh1-L430P bands on a western blot, Pfh1-GFP from an *nmt^+^* repressible promoter system was used so that its expression could be turned off by addition of thiamine. The western blot revealed that Pfh1-GFP was not expressed in the presence of thiamine (WT, Figure 2B), whereas Pfh1-L430P expression was seen in three of three independent clones (1–3, Figure 2B).

Separation of function alleles *pfh1-nuc* and pfh1-mt* produce protein that localizes to the nucleus and mitochondria, respectively [39]. To determine whether *pfh1-L430P* can complement either the nuclear or the mitochondrial helicase function, strains with *pfh1-nuc* or pfh1-mt* allele were used for a second complementation experiment (Table S3). Similar to the experiment in Figure 2A, *pfh1-nuc* or pfh1-mt* strains contained WT *pfh1^+^* flanked by loxP sites and *pfh1-L430P* expressed under its endogenous promoter at the *leu1-32* locus. After the transformation of Cre recombinase, few Ura^+^ transformants were observed (Figure 2C, top row). In contrast, the transformation control (*cre^−^*) yielded many Ura^+^ colonies (Figure 2C, bottom row). From this data, it can be concluded that the point mutation *pfh1-L430P* cannot complement the essential function of Pfh1 in either the nucleus or the mitochondria.

## Discussion

*BRCA1* is a classic tumor suppressor gene in that loss of the wild-type allele (loss of heterozygosity, LOH) is required for tumorigenesis in germline mutation carriers. In sporadic breast tumors, allelic loss of *BRCA1* is common [10]–[12]. Knudson's model would have predicted that in at least a proportion of these cases, *BRCA1* is inactivated by a somatically acquired mutation. Contradicting this hypothesis is the observation that somatic BRCA1 mutations are exceedingly rare in sporadic carcinomas. However, *BRCA1* message and protein are often decreased in sporadic breast and ovarian cancers [13], [14], [40], [41]. In some cases, BRCA1 is down-regulated by aberrant methylation. Methylation of the BRCA1 promoter occurs in 11–14% [42]–[44] of sporadic breast cancers and in 5–31% [42], [44]–[46] of ovarian cancers and is often associated with LOH [42], [47]. However, other mechanisms responsible for loss of BRCA1 in sporadic disease remain to be determined.

*Alu*-mediated aberrant homologous recombination contributes to loss of BRCA1 in a significant proportion of inherited disease. Importantly, this mechanism may explain some of the allelic loss of BRCA1 observed in sporadic disease. Thus we have taken a genomewide approach to identify genes that suppress *Alu*-mediated recombination in yeast with the knowledge that this screen would fail to reveal any human genes involved in suppressing *Alu*-mediated recombination for which no human homologs are present in yeast. A functional screen of the complete set of yeast deletion strains in the BY4742 background identified twelve strains with *Alu*-mediated recombination rates greater than 1.5 fold that of the wild-type strain (Table 1). To confirm that the 5-FOA^R^ phenotype of the newly identified suppressors of *Alu*-mediated recombination was the result of deletion of the given open reading frames (ORFs), their counterparts in the BY4741 background were analyzed. Only 4 of the 12 strains were validated in BY4741, raising the possibility that the increased rate of *Alu*-mediated recombination in the other 8 strains was not related to deletion of the indicated gene. Of the 4 validated strains, mutation rates for *arg3*, *tsa1*, and *rrm3* were significant when compared to the respective wild-type rate. While the mutation rate between *oma1* in BY4741 and BY4742 was consistent, suggesting that this gene has a role in suppressing *Alu*-mediated recombination, the corresponding p-values were not significant when compared to the respective wild-type strain.

Deletion of TSA1, a thioredoxin peroxidase, has been shown to increase the rate of both spontaneous mutation as well as gross chromosomal rearrangement (GCR) [48], [49]. The relative rate of *tsa1*-permissive GCR is similar to that for *Alu*-mediated recombination (7 and 3.34 [49], and this study, respectively). Together these results indicate the importance of this gene in preventing a broad spectrum of types of genomic instability. The human homologs of TSA1 are the four member of the peroxiredoxin (PRDX) family of antioxidant enzymes which reduce hydrogen peroxide and alkyl hydroperoxides. Homozygote Prdx1^−/−^ mice knockouts develop hemolytic anemia and several malignant cancers including epithelial and mesenchymal tumors such as hepatocellular carcinoma, fibrosarcoma, osteosarcoma, islet cell adenomas, and adenocarcinomas of the lung and breast [50]. Heterozygote Prdx1^+/−^ mice also show increased frequency of hemolytic anemia and malignant cancer.

In contrast, ARG3, an ornithine carbamoyltransferase involved in the biosynthesis of arginine [51] and its human homolog OTC (ornithine transcarbamylase) have not been priorly identified as having a role in maintaining genomic stability. In addition, this protein has not been associated with cancer.

The final human homolog validated in this screen, RRM3, was first identified as a suppressor of recombination in ribosomal DNA (rDNA) [52]. *S. cerevisiae* RRM3 and its paralog, PIF1, belong to the super family IB of 5′-to-3′ directed DNA helicases. The Pif1 family helicases are defined by seven highly conserved helicase signature motifs, three motifs that are shared with *E. coli* RecD and in eukaryotes, and a highly conserved 21-residue Pif1 family signature sequence located between motifs II and III [53]. Mouse and human PIF1 proteins immuno-precipitate with telomerase activity and TERT, the catalytic subunit of telomerase [54]. There is also some data suggesting that, like yeast Pif1, human PIF1 may inhibit telomerase activity *in vivo* and *in vitro* [38].

To determine whether variation in the human homologs of yeast genes that suppress *Alu*-mediated recombination modify the effect of *BRCA1* in mutation carriers, we determined the sequence of these genes in a series of *BRCA1* mutation carriers who had breast cancer and/or ovarian cancer at particularly early ages. Of the eleven variants identified in *BRCA1* mutation carriers, two were identified in controls, and eight of the remaining mutations were not predicted to be damaging by internal alignment programs. Thus, the variants identified in these candidate genes do not appear to contribute to particularly early onset of disease in *BRCA1* mutations carriers.

Numerous genomewide studies have been conducted analyzing LOH in sporadic breast cancers to reveal foci of potential tumor suppressor genes. Recent studies have employed higher resolution array-based CGH (aCGH) showing the enormous complexity of breast cancer genomes. These studies have consistently reported the same large regions of loss (8p, 9p, 13q, 16q) [55]–[58]; the number and identity of tumor suppressor genes that contribute to sporadic breast cancer remains largely unknown. Interestingly, high resolution mapping of regions of losses with frequencies of >30% included 1p32.1-p31.1, which contains *OMA1*, 1p36.33-p34.2, which is very close to *PRDX1*, 10q25.3-qtel, which contains *PRDX3*, 15q21.3-q24.3, which contains *PIF1*, and 11q14.3-qtel, which includes *ATM* [59]. In the present study, LOH analysis of the human homolog suppressors of *Alu*-mediated recombination in 25 grade 3 invasive ductal carcinomas revealed LOH at 26 of 181 loci (14%), among informative cases. LOH frequencies among the chromosomal regions varied from 0% to 30% (Table 1 and Figure S2). For all genes displaying LOH, the retained allele was sequenced to identify inactivating mutations; however, none were identified in this series.

A significant proportion of high-risk breast cancer families are not explained by mutations in known genes, indicating that still unidentified genes may explain cancer risk in these families. To determine if variation in the human homologs of the yeast mutator candidate genes contributes to increased breast cancer risk in high-risk families, a cohort of Ashkenazi Jewish probands was sequenced for each of the human homologs. Three unreported variants in candidate genes were identified in this population (Table 1 and Table S8). Of these, only variant L319P in PIF1 was reported to be damaging by PolyPhen and not tolerated by SIFT. While this variant was not observed in 368 controls nor has it been reported in dbSNP or the 1000 Genomes Project, we identified it in 2 out of 844 breast cancer cases of Ashkenazi Jewish ancestry. The leucine at amino acid 319 is completely conserved within the PIF1 family of DNA helicases and is located within the putative Pif1family signature motif located between motifs II and III [53]. Given that it is predicted to be within a helical domain (PredictProtein and PSIPRED), substitution of the five-membered chemical ring of proline from a linearly structured leucine likely disrupts protein structure. However, given the number of Ashkenazi Jewish controls evaluated for L319P we cannot exclude the possibility that this allele may be a rare PIF1 allele limited to this population.

Snow et al., reported that Pif1 (−/−) mice are viable at expected frequencies and displayed no visible abnormalities or increases tumor burden. These results seem to contradict those present here suggesting that loss of PIF1 function may contribute to breast carcinogenesis. However, for many genes it is well known that findings in mouse mutants cannot necessarily be extrapolated to humans. For example, early attempts to develop mouse models of BRCA1-linked breast cancer were unsuccessful (reviewed in [60]). Early embryonic lethality precluded tumor development in Brca1 (−/−) mice. Surprisingly, conventional null or hypomorphic Brca1 alleles revealed lack of tumor formation in heterozygous mice. However, homozygous mice with certain hypomorphic Brca1 alleles can survive to adulthood and display an increased susceptibility to a range of tumors, including mammary carcinomas [61]. Tumors can also be induced by conditional inactivation of Brca1 in breast epithelial cells through cre/loxP-mediated recombination [62]. Inactivation of Brca1 alone in murine ovarian surface epithelium resulted in an increased accumulation of premalignant changes, but no tumor formation [63]. Importantly, somatic loss of both Brca1 and p53 resulted in the rapid and efficient formation of highly proliferative, poorly differentiated estrogen receptor-negative mammary tumors that closely mimic human BRCA-mutated breast cancers with basal-like phenotypes [64] suggesting that other genetic events contribute to tumorigenesis. Approximately 50% of familial breast cancer remains unresolved- that is disease cannot be explained by loss of function mutations in known breast cancer genes. Thus other genes are worthy of in-depth genomic analysis in unresolved families regardless of their associated mouse phenotype.

To determine if additional PIF1 variants impact breast cancer risk, we determined the complete PIF1 coding sequence in a series of 400 additional high risk breast cancer probands largely of European ancestry. Of the variants identified in this series, S223T, P357L, and R592C are potentially deleterious (Tables 1 and 2). In *S. cerevisiae Rrm3*, threonine occupies the position corresponding to human S233 thus it is unlikely that this variant impacts human PIF1 function; however, the amino acid is immediately adjacent to conserved helicase motif I (a nucleotide binding motif also known as “the Walker A box”) and as such could affect ATPase activity. The human proline at position 357 is adjacent to motif III and completely conserved within the family. Thus it is possible the nonconservative amino acid change P357L contributes to protein destabilization. R592C is a relatively conserved amino acid position (R or Q in humans, mice, both yeast Pif helicases, and *E. coli* RecD). While this substitution results in an amino acid with a smaller side chain, it is conservative in terms of hydrophilicity. However, in yeast, mutations in this region tend to disrupt helicase activity but not ATP binding or hydrolysis thereby impairing the ability of the protein to couple conformational changes caused by ATPase activity to DNA unwinding [65]. Finally, PIF1 variant P109L, which was found in a BRCA1 mutation carrier with particularly early onset breast cancer as well as in 1 of 198 controls, is predicted to be deleterious. Although this proline resides over 100 residues upstream of motif I, it is completely conserved from yeast to humans.

The Pif1 family of 5′ to 3′ DNA helicases is conserved from yeasts to humans. While the Pif1 helicase function is dispensable in *S. cerevisiae* and mouse, the *S. pombe* Pfh1 is essential in both mitochondria and nuclei [39]. The results shown here demonstrate that the *pfh1-L430P* allele does not provide the essential activity of Pfh1 (Figure 2A). Since Pfh1-L430P is expressed, its failure to complement is not due to misfolding and degradation of the mutant protein (Figure 2B). Pfh1-L430P does not complement Pfh1 helicase activity in either the nucleus or the mitochondrial (Figure 2C). Therefore, we conclude the lethality of cells expressing Pfh1-L430P is due to loss of helicase function in both the nucleus and mitochondria.

Here we report the systematic analysis of the complete set of yeast gene deletion mutants to identify genes required for preventing *Alu*-mediated aberrant homologous recombination events, providing a global view of these nonessential genes in maintaining genome stability. We identified both previously known suppressors of chromosomal rearrangements as well as a number of novel genes. We provide genetic and functional evidence that a rare, loss of function variant in the helicase PIF1 may elevate breast cancer risk. Finally, although the primary aim of this research was to identify novel genes involved in genomic rearrangement at the *BRCA1* locus, the genes identified in this screen may also contribute to chromosomal rearrangement at other loci. As such, they should be considered as candidate genes capable of facilitating cancer-inducing deletions, duplications, translocations, and splice variations in other tumor types.

## Supporting Information

Figure S1 **Scoring of −Leu/+5FOA plates with cells transformed with pAUA.** Four rows (48 wells) from each yeast deletion transformation plate were streaked onto −Leu/+5FOA media. This plate is an example. 5-FOA^R^ scoring is noted to illustrate scores of 0 (0 colonies), 1 (1–5 colonies), 2 (6–10 colonies), 3 (11–15 colonies), 4 (16–34 colonies), and 5 (≥35 colonies). The wild-type strain had a score of 1.
(TIF)

Figure S2 **LOH in sporadic breast tumors.** Twenty-five sporadic breast tumors and matched normal DNA were tested for loss of heterozygosity (LOH). Two markers closely flanking each gene were tested and loss of heterozygosity at either marker indicated LOH at the gene. The percentage of LOH at each gene is indicated below the dot plot.
(TIF)

Table S1 **Oligonucleotides used in the process of creating and sequencing pAUA.**
(DOCX)

Table S2 **Microsatellite markers used for LOH analysis in sporadic breast tumors.**
(DOCX)

Table S3 ***S. pombe*** **strains.**
(DOC)

Table S4 **Results of yeast deletion strain 5FOA screen with plasmid pAUA.**
(DOCX)

Table S5 ***Alu*** **-mediated recombination rate for yeast deletion strains (BY4741) transformed with pAUA.**
(DOCX)

Table S6 **Human Homologs of Candidate Suppressors of** ***Alu*** **-mediated recombination.**
(DOCX)

Table S7 **Novel variants in candidate suppressors of** ***Alu*** **-mediated recombination in** ***BRCA1*** **mutation carriers presenting with exceptionally early breast and/or ovarian cancer.**
(DOCX)

Table S8 **Novel variants in candidate suppressors of** ***Alu*** **-mediated recombination in high risk Ashkenazi Jewish breast cancer families.**
(DOCX)
