# Supplementary figures and images for: A Genomewide Screen for Suppressors of Alu-Mediated Rearrangements Reveals a Role for PIF1
Source: PLoS One. 2012 Feb 9;7(2):e30748. doi: 10.1371/journal.pone.0030748 (PMC3276492; doi:10.1371/journal.pone.0030748)

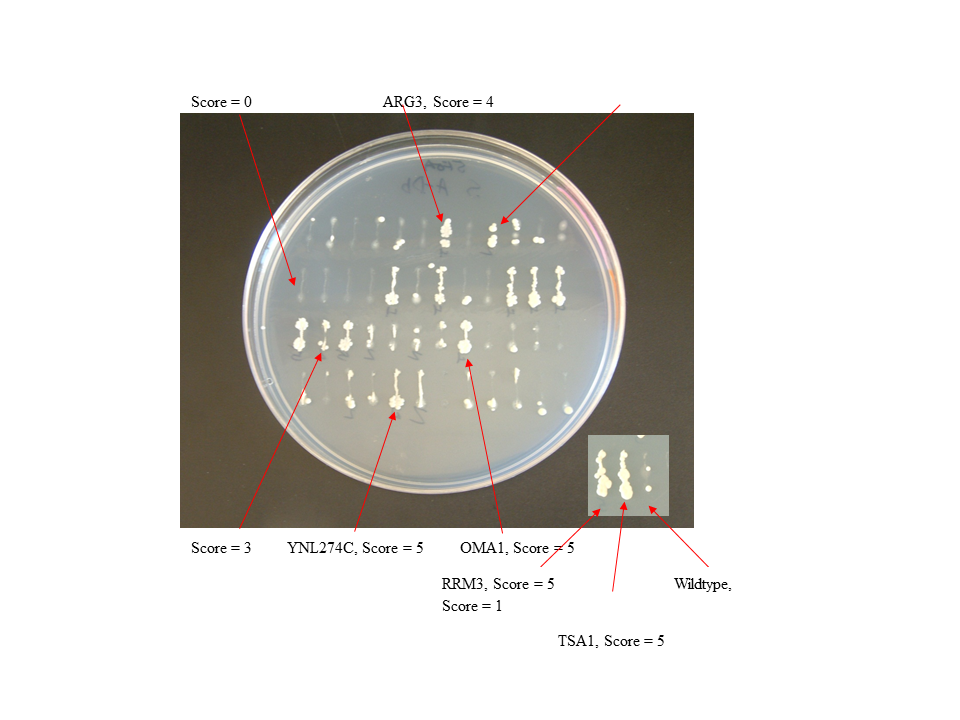

Supplement: Figure S1 — Scoring of −Leu/+5FOA plates with cells transformed with pAUA. Four rows (48 wells) from each yeast deletion transformation plate were streaked onto −Leu/+5FOA media. This plate is an example. 5-FOAR scoring is noted to illustrate scores of 0 (0 colonies), 1 (1–5 colonies), 2 (6–10 colonies), 3 (11–15 colonies), 4 (16–34 colonies), and 5 (≥35 colonies). The wild-type strain had a score of 1. (TIF) [file pone.0030748.s001.tif]

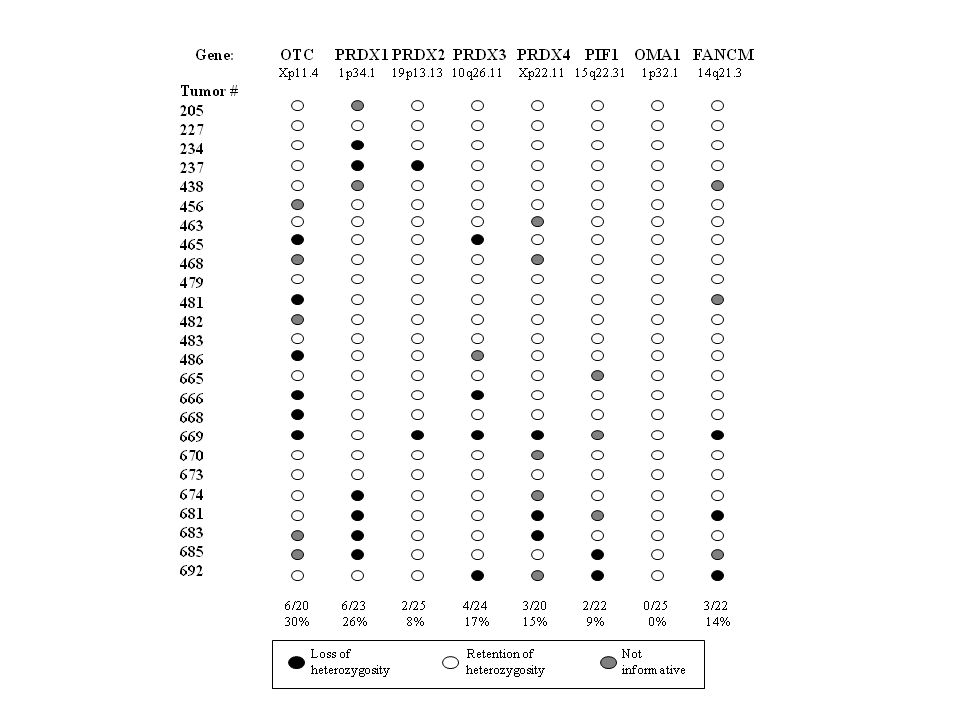

Supplement: Figure S2 — LOH in sporadic breast tumors. Twenty-five sporadic breast tumors and matched normal DNA were tested for loss of heterozygosity (LOH). Two markers closely flanking each gene were tested and loss of heterozygosity at either marker indicated LOH at the gene. The percentage of LOH at each gene is indicated below the dot plot. (TIF) [file pone.0030748.s002.tif]
