# Supplementary material for: A Genomewide Screen for Suppressors of Alu-Mediated Rearrangements Reveals a Role for PIF1
Source: PLoS One. 2012 Feb 9;7(2):e30748. doi: 10.1371/journal.pone.0030748 (PMC3276492; doi:10.1371/journal.pone.0030748)
Supplement: Table S1 — Oligonucleotides used in the process of creating and sequencing pAUA. (DOCX) [file pone.0030748.s003.docx]

**Supplementary Table S1. Oligonucleotides used in the process of creating and sequencing pAUA.**

| ***Oligonucleotide*** | ***Sequence (5’ to 3’)*** |
| --- | --- |
| Asc_Nco_Alu_F | GGC GCG CCA TGC CAT GGC CCC TGT AAT TAA TTG TCA TTG TC |
| Asc_Alu_R | GGC GCG CCA TTT GAT AAT CTG GTA TGT TAT C |
| Bam_Alu_F | CGC GGA TCC CTG TAA TTA ATT GTC ATT GTC |
| Bam_Asc_Alu_R | GAC GGA TCC GGC GCG CCA TTT GAT AAT CTG GTA TGT T |
| Nco_Ura_F | CAT GCC ATG GCG GCA TCA GAG CAG ATT GTA C |
| Nco_Ura_R | CTA GCC ATG GCT CCT TAC GCA TCT GTG CGG |
| Ura_Nco_Mutation | GGA TAT CTT GAC TGA TTT TTC GAT GGA GGG CAC AGT TAA GCC G |
| pCR2.1_M13F | GTA AAA CGA CGG CCA G |
| pCR2.1_M13R | CAG GAA ACA GCT ATG AC |
| URA_Nco_Seq2 | CAG TAG ATA GGG AGC CCT TG |
| URA_Nco_Seq3 | CCT TGG TGG TAC GAA CAT CC |
| pRS415_MCS_F | CGA CGG CCA GTG AGC GCG CG |
| pRS415_MCS_R | CAC AGG AAA CAG CTA TGA CC |
