# Supplementary material for: A Genomewide Screen for Suppressors of Alu-Mediated Rearrangements Reveals a Role for PIF1
Source: PLoS One. 2012 Feb 9;7(2):e30748. doi: 10.1371/journal.pone.0030748 (PMC3276492; doi:10.1371/journal.pone.0030748)
Supplement: Table S2 — Microsatellite markers used for LOH analysis in sporadic breast tumors. (DOCX) [file pone.0030748.s004.docx]

**Supplementary Table S2. Microsatellite markers used for LOH analysis in sporadic breast tumors.**

| ***Gene*** | ***Chr loc*** | ***Markers^a^*** | ***Distance from gene*** |
| --- | --- | --- | --- |
| OTC | Xp11.4 | (TTTG)38.28 | Proximal by 112.5 kb |
|  |  | (GATA)37.78 | Distal by 314.3 kb |
| PRDX1 | 1p34.1 | D1S3175 | Proximal by 30.0 kb |
|  |  | (TCTA)45.62 | Distal by 126.6 kb |
| PRDX2 | 19p13.13 | (TTTC)12.93 | Proximal by 160.3 kb |
|  |  | D19S221 | Distal by 194.5 kb |
| PRDX3 | 10q26.11 | (TG)120.75 | Proximal by 166.5 kb |
|  |  | (CCTT)121.09 | Distal by 166.0 kb |
| PRDX4 | Xp22.11 | (CTAT)23.77 | Proximal by 151.6 kb |
|  |  | (AC)23.47 | Distal by 127.9 kb |
| PIF1 | 15q22.31 | (GT)62.89 | Proximal by 3.6 kb |
|  |  | (TG)62.95 | Distal by 49.0 kb |
| OMA1 | 1p32.1 | D1S2700 | Proximal by 104.5 kb |
|  |  | D1S405 | Distal by 137.3 kb |
| FANCM | 14q21.3 | (AT)43.98 | Proximal by 695.2 kb |
|  |  | (TTTA)45.03 | Distal by 286.8 kb |

^a^ marker name written in the D(chromosome#)S(marker #) format with the repeat unit in parentheses followed by the megabase position on the chromosome.
