# Supplementary material for: A Genomewide Screen for Suppressors of Alu-Mediated Rearrangements Reveals a Role for PIF1
Source: PLoS One. 2012 Feb 9;7(2):e30748. doi: 10.1371/journal.pone.0030748 (PMC3276492; doi:10.1371/journal.pone.0030748)
Supplement: Table S3 — S. pombe strains. (DOC) [file pone.0030748.s005.doc]

**Supplementary Table S3. *S. pombe* strains.**

| ***S. pombe* strains** | | |
| --- | --- | --- |
| SA67 | h+ | ade6-M210 leu1-32::pfh1-L430P ura4-D18 his3-D1 pfh1::loxP pfh1^+^ kan MX6 loxP |
| SA68 | h+ | ade6-M210 leu1-32::pfh1-L430P ura4-D18 his3-D1 pfh1::loxP pfh1^+^ kan MX6 loxP |
| SP421 | h+ | ade6-M210 his3-D1 leu1-32 ura4-D18 pfh1::loxP pfh1^+^ kan MX6 loxP |
| SP425 | h+ | ade6-M210 his3-D1 leu1-32::leu^+^ pfh1^+^ ura4-D18 pfh1::loxP pfh1^+^ kan MX6 loxP |
| SA60 | h+ | ade6-M210 leu1-32 ura4-D18 his3-D1 pfh1::ura^+^nmt81 pfh1 GFP |
| SA69 | h+ | ade6-M210 leu1-32::leu^+^ pfh1-L430P ura4-D18 his3-D1 pfh1::ura^+^nmt81 pfh1 GFP |
| SA70 | h+ | ade6-M210 leu1-32::leu^+^ pfh1-L430P ura4-D18 his3-D1 pfh1::ura^+^nmt81 pfh1 GFP |
| SA71 | h+ | ade6-M210 leu1-32::leu^+^ pfh1-L430P ura4-D18 his3-D1 pfh1::ura^+^nmt81 pfh1 GFP |
| SA76 | h+ | ade6-M210 leu1-32::leu^+^ pfh1-L430P ura4-D18 his3-D1::pJKHis-pfh1-mt* pfh1::loxP pfh1 kanMX6 loxP |
| SA78 | h+ | ade6-M210 leu1-32::leu^+^ pfh1-L430P ura4-D18 his3-D1::pJKHis-pfh1-M1A pfh1::loxP pfh1 kanMX6 loxP |
