# Supplementary material for: A Genomewide Screen for Suppressors of Alu-Mediated Rearrangements Reveals a Role for PIF1
Source: PLoS One. 2012 Feb 9;7(2):e30748. doi: 10.1371/journal.pone.0030748 (PMC3276492; doi:10.1371/journal.pone.0030748)
Supplement: Table S4 — Results of yeast deletion strain 5FOA screen with plasmid pAUA. (DOCX) [file pone.0030748.s006.docx]

**Supplementary Table S4. Results of yeast deletion strain 5FOA screen with plasmid pAUA**

| **Total 5-FOA^R^ score** ^a^ | **0-4** | **5-6** | **7** | **8** | **9** | **10** |
| --- | --- | --- | --- | --- | --- | --- |
| **Number of strains** | 2490 | 1403 | 193 | 285 | 213 | 50 |
| **Proportion of strains screened** | 0.54 | 0.30 | 0.04 | 0.06 | 0.05 | 0.01 |

^a^ 5-FOA^R^ score is the sum of each of two individual colony scores for a given deletion strain.
