# Supplementary material for: A Genomewide Screen for Suppressors of Alu-Mediated Rearrangements Reveals a Role for PIF1
Source: PLoS One. 2012 Feb 9;7(2):e30748. doi: 10.1371/journal.pone.0030748 (PMC3276492; doi:10.1371/journal.pone.0030748)
Supplement: Table S5 — Alu -mediated recombination rate for yeast deletion strains (BY4741) transformed with pAUA. (DOCX) [file pone.0030748.s007.docx]

**Supplementary Table S5. *Alu*-mediated recombination rate for yeast deletion strains (BY4741) transformed with pAUA.**

| *Yeast ORF* | *Yeast Gene* | *Mutation Rate x 10^-6^* | *Fold Induction over Wild-type* | *p-value* |
| --- | --- | --- | --- | --- |
| YJL088W | ARG3 | 25.4 | 11.77 | 0.043 |
| YHR031C | RRM3 | 7.57 | 3.51 | 0.031 |
| YML028W | TSA1 | 6.63 | 3.07 | 0.001 |
| YKR087C | OMA1 | 4.04 | 1.87 | 0.287 |
| YNL274C | GOR1 | 2.78 | 1.28 | 0.324 |
| YOR144C | ELG1 | 2.37 | 1.10 | 0.419 |
| YBL088C | TEL1 | 2.27 | 1.05 | 0.455 |
| **Wild-type BY4741** |  | 2.16 | 1.00 |  |
| YBR272C | HSM3 | 2.04 | 0.95 | 0.471 |
| YPR007C | REC8 | 2.01 | 0.93 | 0.458 |
| YPR170C | Orf | 1.41 | 0.65 | 0.184 |
| YHR157W | REC104 | 1.30 | 0.60 | 0.175 |
| YIR002C | MPH1 | 0.91 | 0.42 | 0.079 |
