# Supplementary material for: A Genomewide Screen for Suppressors of Alu-Mediated Rearrangements Reveals a Role for PIF1
Source: PLoS One. 2012 Feb 9;7(2):e30748. doi: 10.1371/journal.pone.0030748 (PMC3276492; doi:10.1371/journal.pone.0030748)
Supplement: Table S6 — Human Homologs of Candidate Suppressors of Alu -mediated recombination. (DOCX) [file pone.0030748.s008.docx]

**Supplementary Table S6. Human Homologs of Candidate Suppressors of *Alu*-mediated recombination.**

| ***Yeast ORF*** | ***Yeast Gene*** | ***Human Homolog(s)*** | ***BLASTp E-value*** | ***Location*** | ***Description*** |
| --- | --- | --- | --- | --- | --- |
| YJL088W | ARG3 | OTC | 2e-67 | Xp11.4 | Orthinine carbamoyltransferase |
| YOR144C | ELG1 | none |  |  |  |
| YML028W | TSA1 | PRDX1 PRDX2 PRDX3 PRDX4 | 2e-65 5e-72 6e-66 2e-62 | 1p34.1 19p13.13 10q26.11 Xp22.11 | Thioredoxin peroxidases |
| YHR031C | RRM3 | PIF1 (C15orf20) | 9e-59 | 15q22.31 | DNA helicase |
| YPR170C | orf | none |  |  |  |
| YKR087C | OMA1 | OMA1 | 2e-28 | 1p32.1 | Zinc metallopeptidase |
| YIR002C | MPH1 | FANCM | 2e-89 | 14q21.3 | ATP-dependent RNA helicase |
| YBR272C | HSM3 | none |  |  |  |
| YHR157W | REC104 | none |  |  |  |
| YPR007C | REC8 | none |  |  |  |
| YNL274C | GOR1 | GRHPR | 7e-34 | 9p13.2 | glyoxylate reductase/ hydroxypyruvate reductase |
| YBL088C | TEL1 | ATM | 4e-93 | 11q22.3 | Serine-protein kinase |
