# Supplementary material for: A Genomewide Screen for Suppressors of Alu-Mediated Rearrangements Reveals a Role for PIF1
Source: PLoS One. 2012 Feb 9;7(2):e30748. doi: 10.1371/journal.pone.0030748 (PMC3276492; doi:10.1371/journal.pone.0030748)
Supplement: Table S7 — Novel variants in candidate suppressors of Alu -mediated recombination in BRCA1 mutation carriers presenting with exceptionally early breast and/or ovarian cancer. (DOCX) [file pone.0030748.s009.docx]

**Supplementary Table S7. Novel variants in candidate suppressors of *Alu*-mediated recombination in *BRCA1* mutation carriers presenting with exceptionally early breast and/or ovarian cancer.**

| ***Gene*** | ***Heterozygous Mutation*** | ***# of families with mutation*** | ***Frequency in controls*** | ***SIFT del?^a^ align.^b^*** | | ***PolyPhen Score Δ ^c^ prediction ^d^*** | | ***1000 Genomes*** | ***dbSNP*** | ***Secondary Structure^e^ Prediction Confidence*** | |
| --- | --- | --- | --- | --- | --- | --- | --- | --- | --- | --- | --- |
| PRDX3^f^ | L181H | 1 | 0 / 200 | 0.17 | 1.00 | 0.561 | benign | Not present | Not present | Strand | 4 |
| PRDX4 | IVS3+3C/T | 1 | 0 / 200 | N/A |  |  | N/A | Not present | Not present | N/A | N/A |
| PIF1^g^ | V21L | 1 | 0 / 200 | 0.16 | 0.41 | 1.465 | benign | Not present | Not present | Strand | 9 |
|  | P109S | 1 | 1 / 198 | 0.05 | 0.49 | 2.506 | probably damaging | Not present | Not present | Coil | 9 |
| OMA1^h^ | D365N | 1 | 0 / 197 | 0.07 | 0.72 |  | possibly damaging | Not present | D365Y; rs77980955 | Coil | 5 |
| FANCM^i^ | T176M | 2 | 1 / 198 | 0.09 | 0.98 | 1.495 | benign | T176I | T176I ^j^; rs77374493 | Helix | 1 |
|  | C182S | 1 | 0 / 198 | 0.79 | 0.98 | 1.437 | benign | Not present | Not present | Coil | 1 |
|  | N689S | 1 | 0 / 200 0 / 70 ^l^ | 0.57 | 0.60 | 0.307 | benign | Not present | Not present | Coil | 2 |
|  | Q1730P | 1 | 0 / 196 | 0.12 | 0.15 | 1.882 | possibly damaging | Q1730Q | Q1730Q ^k^; rs7142192 | Helix | 1 |
|  | I1742V | 5 | 8 / 196 8 / 94 ^l^ | 1.00 | 0.15 | 0.088 | benign | Not present | Not present | Coil | 5 |
|  | V2014A | 1 | 0 / 198 | 0.04 | 0.13 | 1.295 | benign | Not present | Not present | Coil | 9 |

^a^ SIFT prediction probability of deleterious allele (<0.05 is deleterious)

^b^ SIFT alignment score (1.00 is highest)

^c^ PolyPhen Position-Specific Independent Counts (PSIC) profile score difference (large values may indicate that the studied substitution is rarely or never observed in the protein family)

^d^ PolyPhen prediction

^e^ Predicted secondary structure using PSIPRED (confidence level 0 = low; 9 = high)

^f^ PRDX3 gi for SIFT prediction is 47496635; protein identifier for PolyPhen prediction is P30048

^g^ PIF1: gi for SIFT prediction is 82546872; protein identifier for PolyPhen prediction is Q330H5

^h^ OMA1: gi for SIFT prediction is 15277739; protein identifier for PolyPhen prediction is Q96E52

^i^ FANCM: gi for SIFT prediction is 78099254; protein identifier for PolyPhen prediction is Q8IYD8

^j^ average heterozygote = 0.027

^k^ average heterozygote = 0.024

^l^ Tested Ashkenazi Jewish controls
