# Supplementary material for: A Genomewide Screen for Suppressors of Alu-Mediated Rearrangements Reveals a Role for PIF1
Source: PLoS One. 2012 Feb 9;7(2):e30748. doi: 10.1371/journal.pone.0030748 (PMC3276492; doi:10.1371/journal.pone.0030748)
Supplement: Table S8 — Novel variants in candidate suppressors of Alu -mediated recombination in high risk Ashkenazi Jewish breast cancer families. (DOCX) [file pone.0030748.s010.docx]

**Supplementary Table S8. Novel variants in candidate suppressors of *Alu*-mediated recombination in high risk Ashkenazi Jewish breast cancer families.**

| ***Gene*** | ***Heterozygous Mutation*** | ***# of families with mutation*** | ***Frequency in controls*** | ***SIFT del.^a^ align.^b^*** | | ***PolyPhen Score Δ ^c^ prediction ^d^*** | | ***1000 Genomes*** | ***dbSNP*** | ***Secondary Structure^e^ Prediction Confidence*** | |
| --- | --- | --- | --- | --- | --- | --- | --- | --- | --- | --- | --- |
| PIF1^f^ | L319P | 3 | 0 / 184 ^h^  0 / 184 ^i^ | 0.00 | 0.99 | 2.139 | probably damaging | Not present | Not present | Helix | 9 |
| FANCM^g^ | H1703R | 2 | 3 / 93 ^h^ | 0.83 | 0.13 | 1.888 | possibly damaging | Not present | Not present | Coil | 4 |
|  | I1742V | 3 | 8 / 94 ^h^  8 / 196 ^i^ | 1.00 | 0.15 | 0.088 | benign | Not present | Not present | Coil | 5 |

^a^ SIFT prediction probability of deleterious allele (<0.05 is deleterious)

^b^ SIFT alignment score (1.00 is highest)

^c^ PolyPhen Position-Specific Independent Counts (PSIC) profile score difference (large values may indicate that the studied substitution is rarely or never observed in the protein family)

^d^ PolyPhen prediction

^e^ Predicted secondary structure using PSIPRED (confidence level 0 = low; 9 = high)

^f^ PIF1: gi for SIFT prediction is 82546872; protein identifier for PolyPhen prediction is Q330H5

^g^ FANCM: gi for SIFT prediction is 78099254; protein identifier for PolyPhen prediction is Q8IYD8

^h^ Ashkenazi Jewish control group

^i^ Caucasian control group
